# Fabrication of Wood-Rubber Composites Using Rubber Compound as a Bonding Agent Instead of Adhesives

**DOI:** 10.3390/ma9060469

**Published:** 2016-06-14

**Authors:** Dongwei Shao, Min Xu, Liping Cai, Sheldon Q. Shi

**Affiliations:** 1 Key Laboratory of Bio-Based Material Science and Technology (Ministry of Education), Northeast Forestry University, Harbin 150040, China; shaodongwei2024@163.com; 2 College of Mechanical Engineering, Jiamusi University, Jiamusi 154007, China; 3 Mechanical and Energy Engineering Department, University of North Texas, Denton, TX 76201, USA; Liping.Cai@unt.edu (L.C.); Sheldon.Shi@unt.edu (S.Q.S.)

**Keywords:** wood fiber, rubber compound, rubber processing technology, panel properties, nonlinear programming

## Abstract

Differing from the hot-pressing method in the manufacturing of traditional wood-rubber composites (WRCs), this study was aimed at fabricating WRCs using rubber processing to improve water resistance and mechanical properties. Three steps were used to make WRCs, namely, fiber-rubber mixing, tabletting, and the vulcanization molding process. Ninety-six WRC panels were made with wood fiber contents of 0%–50% at rotor rotational speeds of 15–45 rpm and filled coefficients of 0.55–0.75. Four regression equations, *i.e.*, the tensile strength (*T*s), elongation at break (*E*b), hardness (*H*a) and rebound resilience (*R*r) as functions of fiber contents, rotational speed and filled coefficient, were derived and a nonlinear programming model were developed to obtain the optimum composite properties. Although the *T*s, *E*b and *R*r of the panels were reduced, *H*a was considerably increased by 17%–58% because of the wood fiber addition. Scanning electron microscope images indicated that fibers were well embedded in rubber matrix. The 24 h water absorption was only 1%–3%, which was much lower than commercial wood-based composites.

## 1. Introduction

As an important renewable natural biomass resource, wood fiber from forest felling and processing residues, and waste wood products, has many desirable properties, such as easy availability, low density, low cost, and high stiffness, for new polymer composite development [1,2,3,4,5,6,7]. However, the drawbacks of wood, such as poor dimensional stability, swelling from moisture absorption and being easily attacked by fungi and insects, limit its wide application [8,9].

As a compensation for the wood drawbacks, rubber has the unique advantages of high compressive performance, low moisture absorption, good damping vibration attenuation, excellent energy absorption, good durability, abrasion resistance, anti-caustic and anti-rot properties [10,11,12]. As a common material in the tire rubber industry [13,14,15], the tire rubber can be used in tough environment and climate conditions due to its ability to withstand high and low temperatures, and high anti-rot properties. Thus, the wood-rubber composites (WRCs) would have multi-functional properties and excellent potential for extended applications. Zhao *et al.* [16] discovered that the sound insulation property of wood/used tire rubber composite panel (WRCP) with commercial urea–formaldehyde (UF) and polymeric methylene diphenyl diisocyanate (PMDI) adhesives was better than that of commercial wood-based particleboards. In addition, the acoustic insulation was significantly improved by the amount of rubber crumbs and PMDI adhesive used in the composite. Sun *et al.* [17] reported that the low natural frequency and high damping ratio due to the existence of large amount of rubber particles in the structure. More energy dissipated in the course of transmission can reduce energy dissipation in the form of sound, thus the vibration and noise were also decreased.

In the recent years, a number of studies have been carried out to manufacture WRCs using wood fibers, adhesives, and waste rubber powder/raw rubber by hot-pressing technology. Vladkova *et al.* [2,3,4,5] used corona-activated conifer wood flour as a filler and phenol-formaldehyde resin to make wood-rubber composites. Song and Hwang [18,19] found that the ratio of wood fiber to rubber particles and amount of methylene diphenyl diisocyanate (MDI) significantly affected panel mechanical properties. MDI could excellently bond the wood fibers and rubber particles together. Panel properties such as Modulus of Rupture (MOR), Modulus of Elasticity (MOE), internal bonding (IB) and Young modulus increased with an increase in resin content. Zhao *et al.* [20] utilized the combination of polymeric methylene diphenyl diisocyanate (PMDI) and urea-formaldehyde (UF) to make wood-rubber composites. The results showed that the board density and some interactions between the experimental variables were significant factors that influenced board mechanical properties. The suggested optimal board manufacturing conditions were about 170 °C pressing temperature, 300 s pressing time, and 1000 g/cm^3^ board density. Xu *et al.* [21,22] improved water resistance and mechanical property of poplar wood-rubber composites by adding microwave-treated waste rubber powder. Through contact angle measurements and microscopic analysis, the results indicated that the surface characteristics were modified after the microwave treatments and the mechanical properties of the wood-rubber composites were improved.

All of the previously described WRCs were fabricated using adhesives to bond wood and rubber using hot-pressing technology. The use of adhesives increased the composite cost and caused environmental concerns due to their toxicity and volatility [23,24,25]. The present research was unique because rubber was used instead of adhesives to bond wood fibers for the composite fabrication. The effects of the fiber contents, rotational speed and filled coefficient on the mechanical properties were explored, and a nonlinear programming model was developed to obtain the optimum composite panel properties.

## 2. Experimental

### 2.1. Materials

Provided by the Wooden Forest Products Co., Ltd. in Harbin, China, the wood fiber (WF) with a length-width ratio of 5:45 and a moisture content of 3%–5%, was used in this study. Supplied by Xingda Rubber Factory in Harbin, China, the rubber compound (RC) contained ingredients of 30% natural rubber (NR), 6% butadiene styrene rubber (SBR), 24% butadiene rubber (BR), 30% carbon black (N330), 1% sulfur, 2.5% zinc oxide, 2% stearic acid, 3% spindle oil, and 1.5% toluene (S.D.).

### 2.2. Sample Preparation

**Figure 1:**
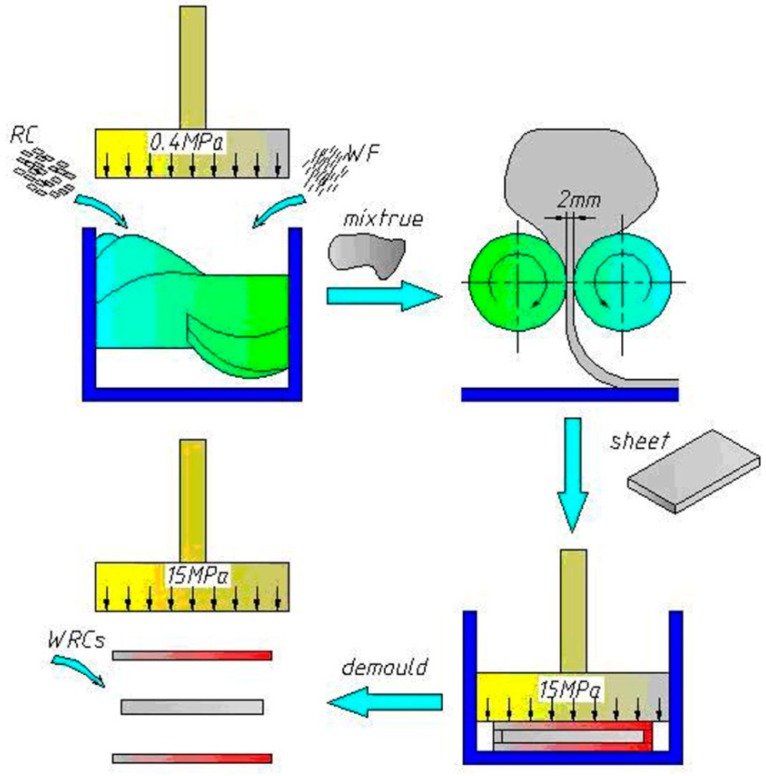
Schematic diagram of the making process of wood-rubber composite (WRC) panel.

The WRCs were manufactured using rubber processing technology, including mixing in the 3L internal mixer, tabletting in the open mill and sulfide forming in a plate vulcanizing machine, in which the mixing is the key step. Mixing quality directly affects the performance of the products. The WRCs with a size of 260 × 260 × 2 mm^3^ (length × width × thickness) were fabricated at a target density of 1.0 g/cm^3^ with a WF content of 0%, 10%, 20%, 30%, 40% or 50% (referred to as F0, F1, F2, F3, F4 and F5, respectively), the main rotor rotational speed ranging from 15–45 rpm, and filled coefficients of 0.55–0.75 in the 3L internal mixer. Six replicate panels were made for each type of composites, resulting in a total of 96 WRCs panels. The process method was conducted in the WRCs sample preparation as shown in Figure 1, and the specific steps are as follows:

1. The sheets of RC were cut into small pieces (about 5 mm^3^) in order to mix them evenly. The small RC pieces were premixed at a mixing temperature of 60 °C for 3 min in the twin rotor mixer (XH-409, Zhuosheng Mechanical Equipment Co., Ltd., Dongguan, China). Then WF was gradually added to the mixer and continued to blend for 5 min.
2. The tabletting process of the mixture was carried out using a two-roll laboratory mill (XH-401A, Zhuosheng Mechanical Equipment Co., Ltd., Dongguan, China) for 3 min. The two-roll miller is consisted of two parallel rolls. The speed ratio between the rolls was 1.2 and gap was 2 mm.
3. After the sheets from the two-roll mill were conditioned in a closed container at a temperature of 23 ± 2 °C for 24 h, the vulcanization was carried out in a plate vulcanizing machine (XH-406B, Zhuosheng Mechanical Equipment Co., Ltd., Dongguan, China) at 160 °C under a pressure of 15 MPa for the optimum curing time (t_90_), which was also called vulcanization molding time.
4. Finally, WRCs were successfully prepared and fetched out from the disassembled mold.

### 2.3. WRC Characterizations

Dumbbell-shaped specimens that were 2 mm thick, 116 mm long, and 25 mm wide (6 mm wide at the narrow portion) were prepared for tensile tests using a Universal Testing Machine (Instron 4505, Norwood, MA, USA) at a crosshead speed of 500 mm/min in accordance with the ISO 37 standard. The hardness of the composite panels was examined by the Shore type A Durometer (JZ-LX-A, Yangzhou, China), in accordance with the standard ISO 7619. The rebound resistance was tested using an Elastic Impact Tester (JZ-6022, Yangzhou, China) in accordance with the ISO 4662 standard, and the tests were performed with a pendulum of 0.5 J potential energy. The scanning electron microscopy (SEM; Quanta 200, FEI Company, Hillsboro, OR, USA) was used to characterize the fracture surface parallel to the thickness direction. The fractured surfaces of the specimens were frozen in liquid nitrogen for 5 min and sputter-coated with gold powder using a SCD-005 sputter coater. The curing characteristics, such as minimum torque replicates (*M*_L_), maximum torque (*M*_H_), scorch time (*t*_s2_), and curing time (*t*_90_), were measured by a No-rotor Rheometer (JZ-6029, Yangzhou, China) according to the standard of ISO6502. The water absorption of the composites was obtained using a 24-h water immersion method at room temperature. The square samples (sizes: 50 × 50 × 2 mm^3^) were cut from the vulcanized samples and dried overnight in a vacuum desiccator. The square samples were weighed (*m*_0_) and placed in distilled water in bottles for 24 h. The liquid on the surface was removed with a filter paper and the specimens were weighed (*m*_1_) right away. The absorption in water (*W*a) was calculated by the following formula:

(1) $$W_{a}=\frac{\left(m_{1}-m_{0}\right)\times 100}{m_{0}}\%$$

where *m*_0_ is the weight of the sample before testing, and m_1_ is the weight of sample after the testing.

**Figure 2:**
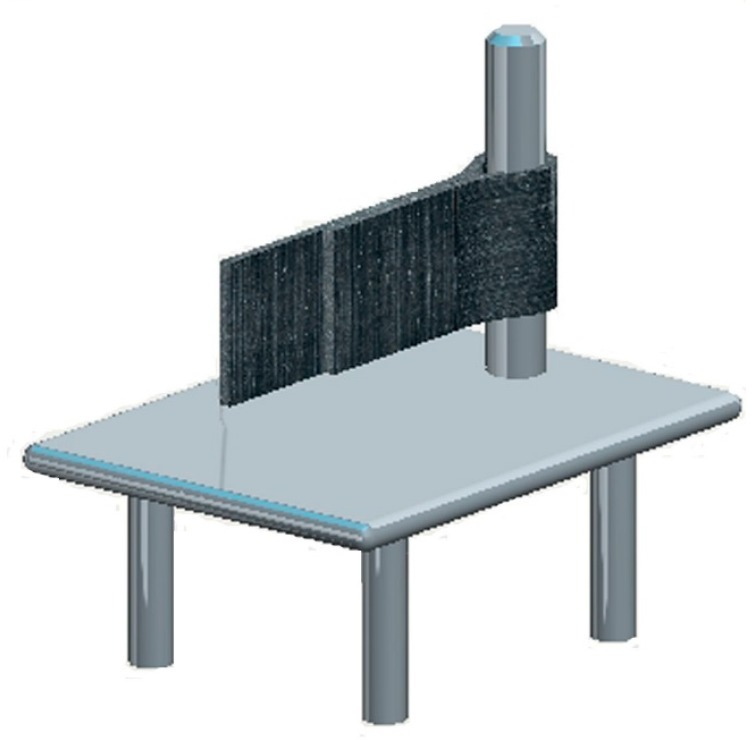
Bending toughness testing device.

The bending toughness tests were carried out using the device (Figure 2) according to the Chinese Chemical Engineering Standard, HG/T 3747.1-2011 “Rubber and plastic floor covering material Part 1”. Samples with a size of 250 mm × 50 mm were bent to 180 degrees along with a vertical metal shaft as shown in Figure 2, and remained in this condition at a temperature of 23 ± 2 °C and a relative humidity of 50% ± 5% for 24 h. Then, the bent outer surface of the sample was visually inspected. Three replicates were completed.

## 3. Results and Discussion

### 3.1. Effect of Wood Fiber Content

**Figure 3:**
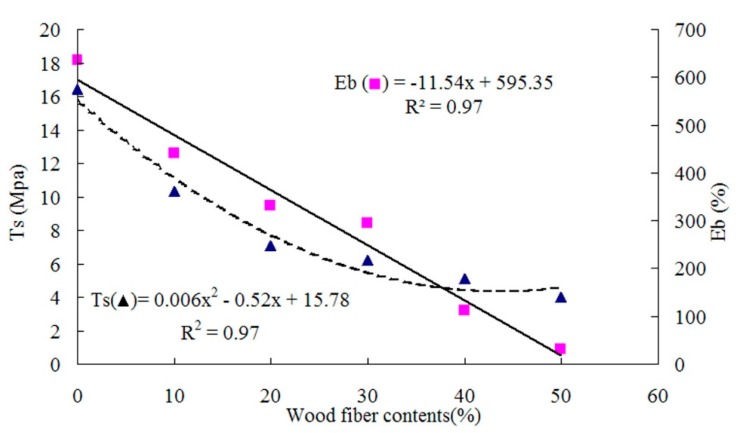
Tensile strength and elongation at break as functions of wood fiber content.

**Figure 4:**
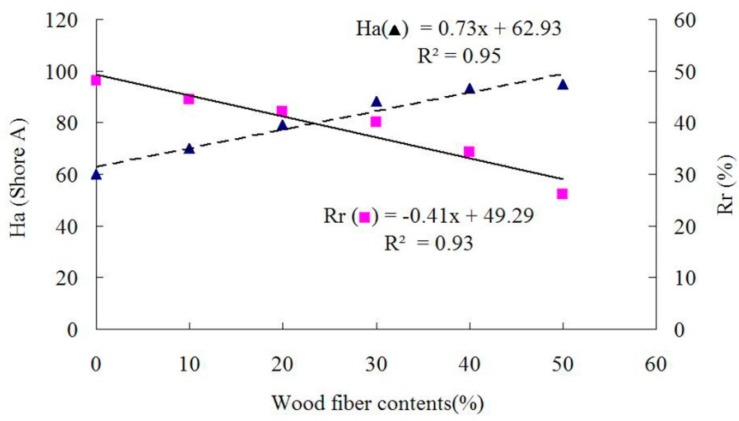
Hardness and rebound resilience as functions of wood fiber content.

The properties of panels prepared with different WF contents at a target panel density of 1 g/cm^3^, rotational speed of 25 rpm, and a filled coefficient of 0.65 are presented in Table 1. The average fiber density was measured to be 0.59 g/cm^3^, and the volume of each composite was 135.2 cm^3^. Based on the different fiber content, the fiber volume fraction for each type of composite was calculated and is listed in Table 1. As shown in Figure 3 and Figure 4, the elongation at break (*E*b), hardness (*H*a) or rebound resilience (*R*r) was a linear function of WF content, respectively, while the relationship between WF content and the tensile strength (*T*s) was polynomial. The bending toughness testing results are presented in Table 1. It was indicated that, when the fiber contents were lower than or equal to 30%, no defect was observed on the sample outer surface, indicating that the bending toughness was perfect. When the fiber contents were over 30%, some cracks were detected, which can be reasoned to be due to too many added fibers having reduced the bending toughness of the samples.

The addition of WF significantly (*p* < 0.05) increased the *H*a values of WRCs. The average *H*a values of WRCs were increased by 17%–58% due to the WF addition, compared to that of control samples (Table 1). It was found that WF played an important role in reinforcing and improving the hardness and rigidity of the composites. With the increased WF addition, *E*b and *R*r were reduced due to the dependence of *E*b and *R*r on the materials. A similarly reduced trend was observed for *T*s, probably because WF was inconsistently dispersed in the rubber matrix and the stress concentration was increased by the increased WF incorporation. As a result, *T*s was reduced as shown in Figure 3. The similar trends of *T*s, *E*b and *H*a were reported by Vladkova *et al.* [2,3].

### 3.2. Effect of Rotational Speed of Shearing Rotor in Internal Mixer

**Figure 5:**
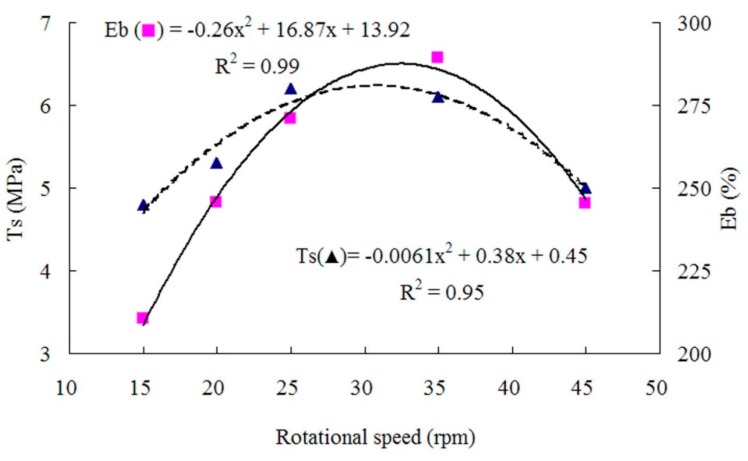
Tensile strength and elongation at break as functions of rotational speed.

**Figure 6:**
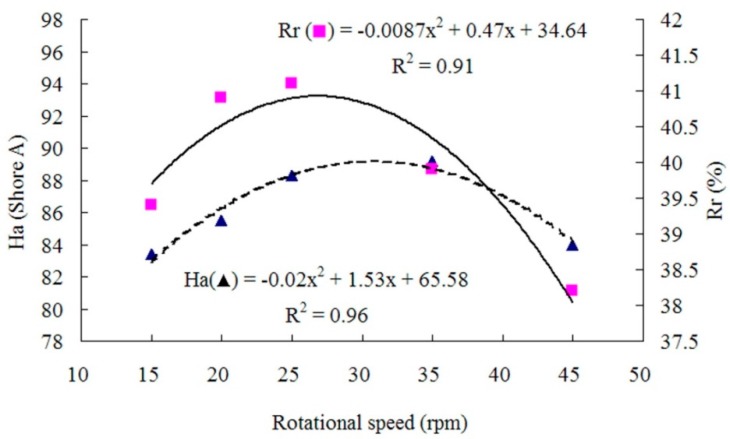
Hardness and rebound resilience as functions of rotational speed.

The properties of panels prepared with different rotor rotational speeds at a target panel density of 1 g/cm^3^, a WF content of 30%, and a filled coefficient of 0.65 are presented in Table 2. As shown in Figure 5 and Figure 6, the relationship between the rotor rotational speed and *T*s, *E*b, *H*a or *R*r was polynomial.

The mechanical properties of the composites were relatively low at the rotational speed of 15 rpm, possibly due to the poor rotor shearing action leading to uneven WF dispersion. First, the mechanical properties increased with the rotational speed, *R*r reached its maximum at about 27 rpm, *T*s reached its maximum at about 30 rpm, and *E*b and Ha reached their maximum at about 33 rpm. The increases in mechanical properties were possibly because the increase in rotor speed strengthened the shearing action to enhance the mixing efficiency and even the WF dispersion. However, when the rotational speed was too high (over 33 rpm), the mixture temperature increased too fast, creating the radial temperature gradient in the mixing chamber and causing scorch. As a result, *T*s, *E*b, *H*a and *R*r decreased when the rotation speed was over 33 rpm.

### 3.3. Effect of Filled Coefficient in Internal Mixer

**Figure 7:**
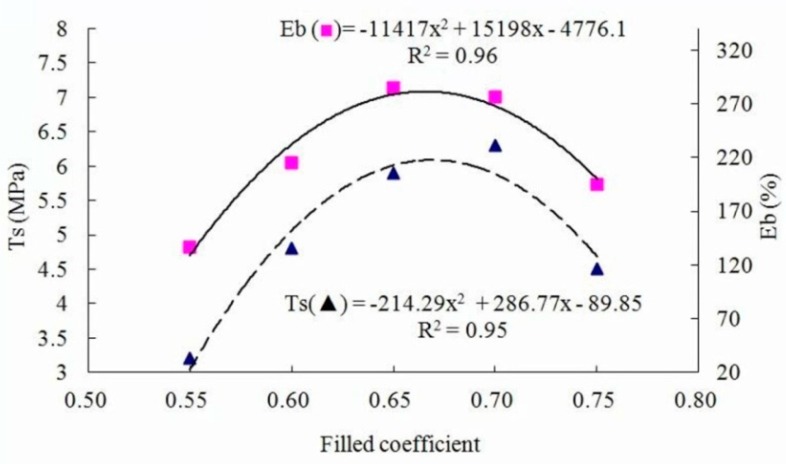
Tensile strength and elongation at break as functions of filled coefficient.

**Figure 8:**
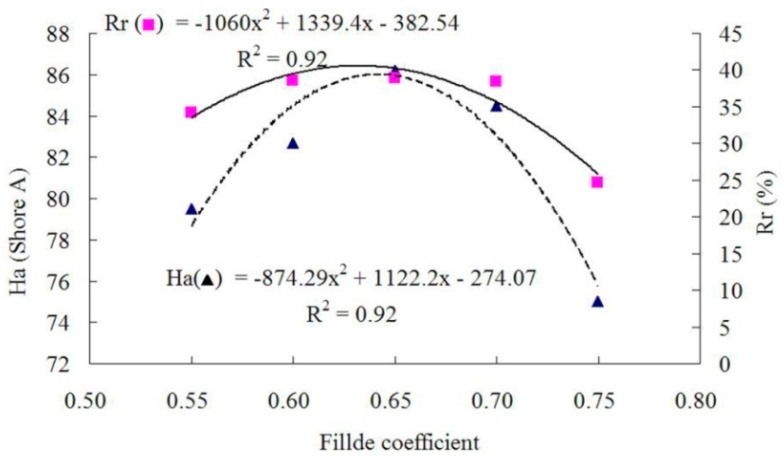
Hardness and rebound resilience as functions of filled coefficient.

The properties of panels prepared with different filled coefficients at a target panel density of 1 g/cm^3^, a WF content of 30%, and a rotor rotational speed of 25 rpm are presented in Table 3. As shown in Figure 7 and Figure 8, the relationships between the filled coefficient and *T*s, *E*b, *H*a or *R*r was polynomial.

With an increase in filled coefficient from 0.55 to 0.75, the mechanical properties of the composites first increased, and then decreased. *T*s and *E*b reached their maximum values at the filled coefficient of about 0.68, and *H*a and *R*r reached maximum at about 0.65, which was possibly because the increase in filled coefficient made the gap in the mixing chamber smaller, resulting in an increase in shear force and the heat transfer area. The minimum mechanical properties at the filled coefficient of 0.55 could be explained by the too-large gap in the mixing chamber. The bonding strength between the rubber and wood fibers was reduced because the too-large gap could not sufficiently shear the fibers and rubber particles. When the filled coefficient increased over 0.68, wood fibers could not be evenly dispersed into the rubber due to the too-small gap in the mixing chamber. As a result, the axial flow and circumferential flow of the extrusion were not sufficient, and then the mechanical properties of the composites were reduced.

### 3.4. Nonlinear Regression

Based on the experimental data (Table 1, Table 2 and Table 3), four regression equations (*T*s, *E*b, *H*a and *R*r, as functions of fiber content, rotational speed and filled coefficient, were obtained as follows (Equations (2)–(5)):

(2) $$Ts=-119.85-0.23x_{1}+377.27x_{2}-283.90{\left(x_{2}\right)}^{2}+0.55x_{3}\;–0.0092{\left(x_{3}\right)}^{2}$$

$$\;R^{2}=0.87$$

(3) $$Eb=-3768.40-11.35+12461x_{2}-9311.60{\left(x_{2}\right)}^{2}+13.04x_{3}\;-0.19{\left(x_{3}\right)}^{2}$$

$$\;R^{2}=0.97$$

(4) $$Ha=-298.07+0.74x_{1}+1091.90x_{2}-851.02{\left(x_{2}\right)}^{2}+0.73x_{3}-0.01{\left(x_{3}\right)}^{2}\;$$

$$\;R^{2}=0.94$$

(5) $$Rr=-290.77-0.38x_{1}+1098.80x_{2}-874.95{\left(x_{2}\right)}^{2}-0.23x_{3}+0.0033{\left(x_{3}\right)}^{2}\;$$

$$\;R^{2}=0.88$$

where $x_{1}$ is the WF content (%); $x_{2\;}$ is filled coefficient in internal mixer; $x_{3}$ is the rotational speed of main rotor in mixing chamber, and; R is the correlation coefficient of each regression equation.

Using these four equations, the *T*s, *E*b, *H*a and *R*r of the panel can be predicted based on production conditions such as the WF content, the filled coefficient in the internal mixer, and the rotational speed of the main rotor in the mixing chamber.

### 3.5. Optimization

Due to the relationship between the independent variables ($x_{1},x_{2},x_{3}$) and dependent variables (*T*s, *E*b, *H*a and *R*r), it is necessary to study the best process conditions of the composite material plate fabrication. In cases where maximum Ha is desired for the panels, the standard of the chemical industry (Chinese Standard HG/T 3747.1-2011, rubber and plastic floor material, Part 1, rubber floor) requires minimum *T*s, *E*b, *H*a and *R*r of 0.3 MPa, 40%, 75 Shore A and 38%, respectively. The following optimization model (Equations (6)–(9)) was established through nonlinear programming:

(6) $$\mathrm{Max}\;Ha\left(x\right)=-298.07+0.74+1091.90x_{2}-851.02{\left(x_{2}\right)}^{2}+0.73x_{3}-0.01{\left(x_{3}\right)}^{2}\;$$

Subject to:

(7) $$-119.85-0.23x_{1}+377.27x_{2}-283.90{\left(x_{2}\right)}^{2}+0.55x_{3}-0.0092{\left(x_{3}\right)}^{2}\geq 0.3$$

(8) $$-3768.4-11.36x_{1}+12461x_{2}-9311.6{\left(x_{2}\right)}^{2}+13.04x_{3}-0.19{\left(x_{3}\right)}^{2}\;\geq 40$$

(9) $$-290.77-0.38x_{1}+1098.80x_{2}-874.95{\left(x_{2}\right)}^{2}-0.23x_{3}+0.003{\left(x_{3}\right)}^{2}\geq 38$$

The experimental condition constraints:

$$0\leq x_{1}\leq 50$$

$$0.55\leq x_{2}\leq 0.75$$

$$15\leq x_{3}\leq 45.$$

The optimal solution for the problem is ($x_{1},x_{2},x_{3}$) = (32, 0.63, 30) with a Max *H*a(*x*) = 87.78. The result indicates that a maximum *H*a of 87.78 Shore A for the panel can be obtained when there is a wood fiber content of 32%, a filled coefficient of 0.63 and a rotational speed of 30 rpm. This makes the *T*s, *E*b and *R*r for the panel equal to 6.08 MPa, 239.29% and 38.02%, respectively, which all exceed the requirements of the standard (HG/T 3747.1-2011).

When mill personnel want to design the production parameters for a special application, the nonlinear programming model can be used as a guideline for the design. In the case of a children’s club floor, the panel material needs the highest tensile strength, proper hardness and elasticity to avoid children being hurt. The nonlinear programming model (Equations (10)–(13)) can be formed as follows:

(10) $$\mathrm{Max}\;Ts\left(x\right)=-119.85-0.23x_{1}+377.27x_{2}-283.90{\left(x_{2}\right)}^{2}+0.55x_{3}-0.0092{\left(x_{3}\right)}^{2}$$

Subject to:

(11) $$-3768.4-11.36x_{1}+12461x_{2}-9311.6{\left(x_{2}\right)}^{2}+13.04x_{3}-0.19{\left(x_{3}\right)}^{2}\geq 40$$

(12) $$-298.07+0.74x_{1}+1091.90x_{2}-851.02{\left(x_{2}\right)}^{2}+0.73x_{3}-0.01{\left(x_{3}\right)}^{2}\;\geq 75$$

(13) $$-290.77-0.38x_{1}+1098.80x_{2}-874.95{\left(x_{2}\right)}^{2}-0.23x_{3}+0.003{\left(x_{3}\right)}^{2}\geq 38$$

The experimental condition constraints:

$$0\leq x_{1}\leq 50$$

$$0.55\leq x_{2}\leq 0.75$$

$$15\leq x_{3}\leq 45.$$

The optimal solution for the problem is ($x_{1},x_{2},x_{3}$) = (15, 0.66, 30) with a max *T*s(*x*) = 10.35, indicating that the maximum *T*s of 10.35 MPa of the panel can be obtained when a wood fiber content of 15%, a filled coefficient of 0.66 and a rotational speed of 30 rpm were utilized. This made *E*b, *H*a and *R*r of the panel equal to 445.83%, 75.01 Shore A and 43.61%, respectively, which met the requirements of the standard (HG/T 3747.1-2011).

### 3.6. Micro-Morphology of the WRCs

**Figure 9:**
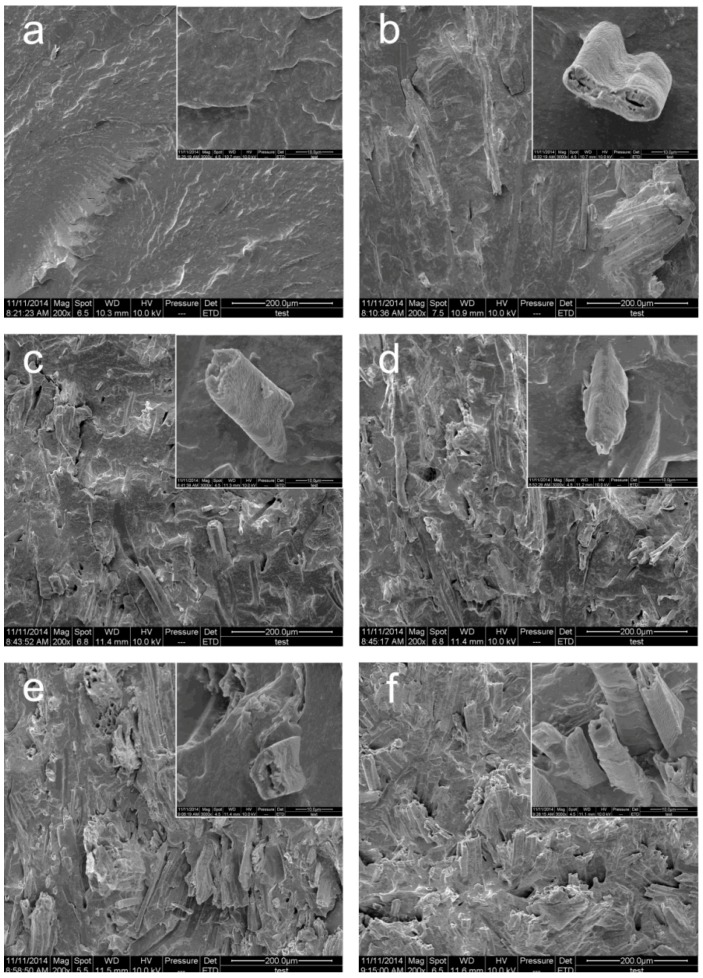
The scanning electron microscope (SEM) photograph of (**a**) pure rubber compound matrix; (**b**) 10 wt % wood fibers; (**c**) 20 wt % wood fibers; (**d**) 30 wt % wood fibers; (**e**) 40 wt % wood fibers; (**f**) 50 wt % wood fibers.

The SEM images of the cryogenically fractured surface of F0, F1, F2, F3, F4 and F5 at WF contents of 0%, 10%, 20%, 30%, 40% and 50%, respectively, are shown in Figure 9a–f. Compared to the control sample F0 (RC) in Figure 9a, WF in the RC matrix could easily be identified in Figure 9b–f. It was observed that the WF concentration on the fracture surface of the samples increased with an increase in WF content. The WF was well wetted by the RC matrix and embedded in the RC matrix. When WF contents were lower than 20%, the fracture surfaces of the samples (Figure 9a,b) appeared to be smooth and flat. When the amount of WF increased (≥20%), the fracture surfaces of the samples (Figure 9c–f) became rougher and gradually transformed to a hummocky fracture, which suggested that WF embedded in rubber matrix hindered the flat extension of the fracture surface. However, on the fracture surface, no separation between the WF and RC was observed, indicating that a good adhesion existed at the interface. The above phenomenon suggested that the WRCs formed a continuous segment at different WF contents.

### 3.7. Cure Characteristics of WRCs

**Figure 10:**
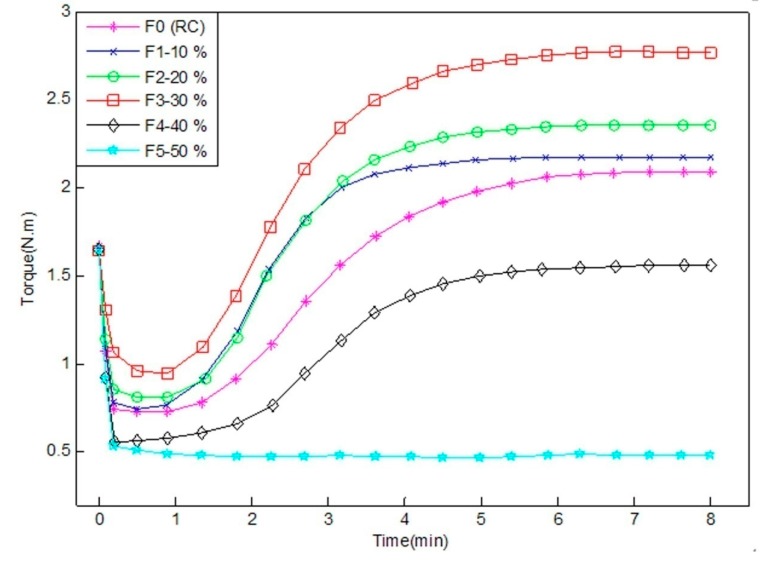
The curing curves of the WRCs.

The processability of the composites is related to their curing characteristics. The curing curves of WRCs at 160 °C are shown in Figure 10. The characteristic parameters of the WRCs cured at 160 °C are shown in Table 4.

As shown in Figure 10, when the WF content varied from 0% to 40%, the torque of the F0, F1, F2, F3 and F4 curves initially decreased, then increased, and finally leveled off. The initial decrease in torque could be due to the softening of the RC matrix, while the increase in torque might be due to the crosslinking of rubber. The leveling off indicated that the curing was completed. When excessive amount of WF was added (>30%), the vulcanization of the WRCs (F4 and F5) became difficult due to the lack of curing agents (the sulfur). The curing curve of the sample F5 shows that it was not vulcanized at all because of its low torque values, as shown in Figure 10.

The minimum torques (*M*_L_) in Table 4 were related to the material flow characteristics. The smaller the *M*_L_ value, the better the liquidity of the un-vulcanized rubber mixture was obtained. The maximum torque (*M*_H_) in Table 4 is a measure of the elastic modulus related to the crosslink density and the stiffness of the materials [26]. The *M*_H_ and *M*_L_ of WRCs increased as the WF content increased from 0% to 30%, then decreased when the wood fiber content continuously increased from 40% to 50%. The increase in M_L_ indicated that the mixture viscosity increased as the WF content increased. As the WF content continually increased in WRCs, the mobility of the macromolecular chains of the rubber was reduced, resulting in more rigid vulcanizations. The increase in *M*_H_ indicated that the stiffness of WRCs increased when the WF content increased from 0% to 30%. This could be attributable to the reinforcing effect of WF. The decrease in M_H_ could be explained by the lack of sulfur. The optimum curing time (*t*_90_) in Table 4 is the vulcanization molding time of the composites. As shown in Table 4, *t*_90_ increased slightly as the WF content increased from 10% to 50%. The longer curing time reflected that the curing rate was slower and the yield was lower. The scorch time (*t*_s2_) in Table 4 presents the times when the rubber starts to vulcanize, *i.e.*, the time from the beginning of the test to the minimum torques (*M*_L_) of the curing curve (Figure 10) rising 0.2 N.m. When the WF content was 30%, the shorter times *t*_s2_ and *t*_90_ in the blends indicated that the cross-linking reaction started earlier with a higher curing rate.

### 3.8. Water Absorption

The 24-h water absorption (*W*a) of WRCs are displayed in Table 5.

The water absorption of the composites increased as WF content increased. The increased water absorption of the composites was due to the hydrophilic nature of the wood fibers. However, for all the composite samples, the water absorption ranged from 1% to 3%, which was a significant reduction compared to the commercial wood-based composites. It was indicated that most wood fibers were encapsulated in the rubber matrixes, which have excellent hydrophobic properties [12].

## 4. Conclusions

The micro-morphology of WRCs indicated that the added WFs were well encapsulated and embedded in RC matrixes. The water adsorptions of WRCs were much lower than that of the commercial wood-based composites. Although the *T*s, *E*b and *R*r of panels were reduced, *H*a was increased considerably by the WF addition. Four regression equations (Equations (2)–(5)) were developed. Given the manufacturing conditions (WF content, filled coefficient and main rotor rotational speed), the WRCs properties can be predicted for different applications. With the nonlinear programming model, we can optimize the manufacturing conditions to obtain the desired *T*s, *E*b, Ha and *R*r properties of composites.

## Figures and Tables

**Table 1:** Properties of the WRCs with different wood fiber contents.

Fiber Contents (%)	Volume Fraction (%)	Tensile Strength (MPa) (*T*s)	Elongation at Break (%) (*E*b)	Hardness (Shore A) (*H*a)	Rebound Resilience (%) (*R*r)	Toughness Tests
0	0	16.4 (1.1) a ^1^	634.1 (19.7) a	60.0 (0) f	48.0 (0) a	No cracks
10	17	10.3 (0.5) b	440.7 (12.8) b	70.2 (0.1) e	44.4 (0.5) b	No cracks
20	33	7.1 (0.2) c	330.1 (13.2) c	79.3 (0.1) d	42.1 (0.3) c	No cracks
30	50	6.2 (0.3) d	293.6 (8.1) d	88.3 (0.2) c	40.0 (0.1) d	No cracks
40	67	5.1 (0.4) e	110.8 (5.7) e	93.2 (0.3) b	34.3 (1.1) e	Fine cracks
50	83	4.0 (0.1) f	31.3 (9.2) f	95.0 (0.1) a	26.1 (0.4) f	Heavy cracks

^1^ Groups with the same letters in each column indicate that there is no statistical difference (*p* < 0.05) between the samples according to the Duncan’s multiple range test. Values in parentheses are standard deviations.

**Table 2:** Properties of the WRCs with different rotational speed of the shearing rotor.

Rotational Speed (rpm)	Tensile Strength (MPa)	Elongation at Break (%)	Hardness (Shore A)	Rebound Resilience (%)
15	4.8 (0.4) c ^1^	210.4 (13.5) d	83.4 (1.2) c	39.4 (0.7) c
20	5.3 (0.1) b	245.5 (15.2) c	85.5 (1.1) b	40.9 (0.5) ab
25	6.2 (0.3) a	270.8 (8.1) b	88.3 (0.2) a	41.1 (0.3) a
35	6.1 (0.4) a	289.3 (5.9) a	89.2 (2.6) a	39.9 (1.5) bc
45	5.0 (0.2) b	245.2 (4.2) c	84.0 (2.1) bc	38.2 (0.7) d

^1^ Groups with the same letters in each column indicate that there is no statistical difference (*p* < 0.05) between the samples according to the Duncan’s multiple range test. Values in parentheses are standard deviations.

**Table 3:** Properties of the WRCs with different filled coefficient.

Filled Coefficient	Tensile Strength (MPa)	Elongation at Break (%)	Hardness (Shore A)	Rebound Resilience (%)
0.55	3.2 (0.4) c ^1^	136.5 (14.3) d	79.5 (0.4) d	34.2 (0.8) b
0.60	4.8 (0.5) b	215.3 (10.6) b	82.7 (0.6) c	38.5 (0.2) a
0.65	5.9 (0.2) a	285.2 (7.5) a	86.2 (0.3) a	38.9 (0.4) a
0.70	6.3 (0.6) a	276.5 (15.1) a	84.5 (0.5) b	38.4 (1.1) a
0.75	4.5 (0.1) b	194.8 (19.5) c	75.0 (0.6) e	24.6 (0.7) c

^1^ Groups with the same letters in each column indicate that there is no statistical difference (*p* < 0.05) between the samples according to the Duncan’s multiple range test. Values in parentheses are standard deviations.

**Table 4:** Cure characteristics of WRCs.

Sample Code	*M*_L_/(N.m)	*M*_H_/(N.m)	*t*_s2_/min	*t*_90_/min
F0	0.68 (0.24) ^1^	2.11 (0.21)	1.97 (0.05)	4.77 (0.21)
F1	0.70 (0.11)	2.17 (0.07)	1.52 (0.21)	3.12 (0.08)
F2	0.79 (0.06)	2.35 (0.13)	1.70 (0.12)	3.65 (0.13)
F3	0.92 (0.32)	2.78 (0.15)	1.57 (0.09)	3.97 (0.14)
F4	0.55 (0.13)	1.56 (0.04)	2.33 (0.11)	4.35 (0.04)
F5	0.46 (0.17)	0.49 (0.16)	0 (0)	5.87 (0.18)

^1^ Values in parentheses are standard deviations. *M*_L_: minimum torques; *M*_H_: maximum torque; *t*_s2_: scorch time; *t*_90_: optimum curing time.

**Table 5:** The water adsorptions of WRCs.

*W*a (%)	F0	F1	F2	F3	F4	F5
Value	1.01 (0.10) ^1^	1.18 (0.08)	1.32 (0.05)	1.52 (0.02)	2.59 (0.04)	2.77 (0.07)

^1^ Values in parentheses are standard deviations.
